# Generation and Characterization of Induced Pluripotent Stem Cells from Mononuclear Cells in Schizophrenic Patients

**DOI:** 10.22074/cellj.2019.5871

**Published:** 2019-02-25

**Authors:** Qing Liu, Jiang Du, Jinyu Fan, Wenqiang Li, Weiyun Guo, Huigen Feng, Juntang Lin

**Affiliations:** 1 Stem Cell and Biotherapy Engineering Research Center of Henan, Xinxiang Medical University, Xinxiang, China; 2 College of Life Science and Technology, Xinxiang Medical University, Xinxiang, China; 3 Henan Key Lab of Biological Psychiatry, The Second Affiliated Hospital of Xinxiang Medical University, Xinxiang, China; 4 College of Biomedical Engineering, Xinxiang Medical University, Xinxiang, China

**Keywords:** Induced Pluripotent Stem Cell, Peripheral Blood Mononuclear Cells, Pluripotency, Reprogramming, Schizophrenia

## Abstract

**Objective:**

Schizophrenia (SZ) is a mental disorder in which psychotic symptoms are the main problem. The pathogenesis of SZ is not fully understood, partly because of limitations in current disease models and technology. The development of induced pluripotent stem cell (iPSC) technology has opened up the possibility of elucidating disease mechanisms in neurodegenerative diseases. Here, we aimed to obtain iPSCs from peripheral blood mononuclear cells (PBMCs) of normal and schizophrenic individuals and analyze the inflammatory response in these iPSCs.

**Materials and Methods:**

In this experimental study, we isolated PBMCs from whole blood of healthy individuals and SZ patients and reprogrammed them into iPSCs by transfection of recombinant lentiviruses that contained Yamanaka factors (Oct4, Sox2, Klf4 and c-Myc). We calculated the numbers of iPSC clones and stained them with alkaline phosphatase (ALP), Nanog, SSEA4, Nestin, Vimentin, and AFP to confirm their efficiency and pluripotency. The iPSCs were analyzed by real-time quantitative polymerase chain reaction (qRT-PCR) for the expressions of inflammatory factors.

**Results:**

iPSCs from schizophrenic patients (SZ-iPSCs) exhibited typical morphology and highly expressed pluripotent markers. These iPSCs retained their normal karyotype and differentiated in vitro to form embryoid bodies (EBs) that expressed markers of all 3 germ layers. However, iPSCs from the SZ-iPSCs group had a weak capacity to differentiate into ectoderm compared to the normal iPSCs (Con-iPSC). An elevated, stronger inflammatory response existed in iPSCs from schizophrenic individuals.

**Conclusion:**

We successfully obtained iPSCs from PBMCs of schizophrenic patients without genetic operation and analyzed the expressions of pluripotent markers and inflammatory factors between the Con-iPSC and SZ-iPSC groups. Taken together, our results may assist to explain the pathogenesis of SZ and develop new strategies for clinical diagnosis and treatment.

## Introduction

Somatic cell reprogramming into a pluripotent state by induced pluripotent stem cell (iPSC) technology not only paves the way for organ regeneration but also provides a powerful tool for studying pathological processes (1, 2). In 2006, Takahashi and Yamanaka (3) were the first to successfully reprogram mouse fibroblasts into iPSCs using retroviral vectors to introduce 4 genes that encode transcription factors (*Oct4, Sox2, Klf4,* and *c-Myc*). iPSCs, like embryonic stem cells (ESCs), can give rise to every other cell type in the body, such as neuron, heart, pancreatic, and liver cells (4). Therefore, iPSCs technology is considered a powerful tool to study the pathological processes of diseases and clinical treatment due to its pluripotency (5). Recently, generation of iPSCs from somatic cells in disease models has become an important method for basic research of pathogenesis (6, 7). 

Schizophrenia (SZ), common mental disorder that usually appears in late adolescence or early adulthood (8), is mainly defined by psychiatric signs that include disorders of delusions, hallucinations, cognition, and emotion (9). SZ has a heavy burden for family and society because of the risk of suicide, lack of medical care, and a higher risk of delusion in schizophrenic patients (10). The pathogenesis of SZ is unclear due to the limitations of current disease models, technology, and clinical approach (11-13). 

Recently, researchers generated iPSCs from human fibroblasts of SZ patients and further induced these iPSCs to form neuron cells. The iPSCs derived neuron cells from SZ patients had lower protein levels of PSD95 compared with the neurons from normal iPSCs, as well as incomplete neuronal development and less junctions (14). Another paper reported that neuron cells from iPSCs that had the *DISC1* mutation, an identified genetic risk factor for SZ, showed defective neuronal synapses and abnormal neuronal gene expression in contrast to normal neurons (15, 16). Hence, iPSCs technology has played an important role in the study and clinical application of SZ (17, 18). In this study, we generated iPSCs from peripheral blood mononuclear cells (PBMCs) of schizophrenic patients and analyzed the morphology, pluripotency, and capacity for differentiation, which would provide choices for the establishment of an SZ model *in vitro*. 

## Materials and Methods

### Isolate and culture of peripheral blood mononuclear cells

The PBMCs were prepared by density gradient centrifugation at room temperature using fresh whole blood both from healthy participants and SZ patients. Then cells were cultured in X-VIVOTM 15 medium (Lonza, Switzerland) supplemented with 1% penicillin/streptomycin. Healthy participants and the family member/legal guardian of SZ patients provided informed consent. This study was approved by the Ethics Review Board at Xinxiang Medical University.

### Generation of induced pluripotent stem cells from peripheral blood mononuclear cells

In this experimental study, we generated human iPSCs from PBMCs by using retroviruses as described previously with some modifications (19). Briefly, after the PBMCs were introduced with Oct4, Sox2, Klf4 and c-Myc, the cells were transferred into a 3 cm^2^ dish covered with Matrigel (BD Biosciences, USA) in X-VIVO^TM^ 15 medium. After 2 days, the cells were reseeded onto dishes with standard iPSC medium, Knockout^TM^ DMEM, that consisted of 20% knockout serum replacement, 1% L-glutamine, 1% nonessential amino acids, 0.1 mM ß-mercaptoethanol, 1% penicillin/ streptomycin, and 10 ng/ml fibroblast growth factor 2 (bFGF, BD Biosciences, USA). We changed the medium every other day until iPSC colonies formed. All cells were cultured in a humidified atmosphere that contained 5% CO_2_ at 37°C. 

### Alkaline phosphatase staining

After infection of the PBMCs, we could observe the iPSC colonies under a fluorescent microscope. To detect alkaline phosphatase (ALP) activity, a cytochemical assay was performed using a Leukocyte Alkaline Phosphatase Kit (Yeasen, China) according to the manufacturer’s protocol. 

### Karyotype analysis

iPSCs were treated for about 45 minutes with Karyo MAX Colcemid (Gibco, USA), harvested, and fixed with methanol:acetic acid (3:1). The cell pellet was washed, resuspended, dropped on a slide, and dried on a hotplate. Cells were stained with Giemsa and the metaphase chromosome number from individual nuclei were counted microscopically (Olympus BX51, Japan, 100X), imaged by CytoVision software, and subjected for G-band karyotyping at Xinxiang Medical University. Karyotypes were described according to the International System for Human Cytogenetic Nomenclature (ISCN) (19). 

### Immunofluorescence staining 

Cells placed on the chamber slides were fixed in 4% paraformaldehyde (PFA, Boster, China) for 15 minutes at room temperature, washed with PBS, and permeabilized with 0.2% Triton X-100 (Sigma, USA) in PBS (PBS-T) for 20 minutes. Cells were incubated overnight in blocking buffer that contained the following primary antibodies: Nanog (1:200; Abcam, USA), SSEA4 (1:200; Abcam, USA), Nestin (1:200; Proteintech, USA), Vimentin (1:200; Abcam, USA), and AFP (1:200; Proteintech, USA) after blocking with 5% fetal bovine serum for 1 hour. Cells were rinsed and primary antibodies detected with appropriate secondary antibodies for 1-2 hours at room temperature in the dark. Then nuclei were counterstained with DAPI. Images were acquired using the Nikon fluorescence microscope and Adobe Photoshop (Adobe Systems) software. 

### Reverse transcription polymerase chain reaction

Total RNA was isolated from cultured cells with the RNeasy Micro Kit (Qiagen, Germany) and reverse transcribed according to the SuperScript III Reverse Transcriptase protocol (Thermo Fisher Scientific). The reverse transcription polymerase chain reaction (RT-PCR) was performed using PrimeSTAR^®^ MAX DNA Polymerase (Takara, Japan).

### *In vitro* differentiation

The iPSCs were plated in ultra-low adhered six-well plates with DMEM/F12 medium that contained 20% KnockoutTM serum replacement, 1% L-glutamine, 1% nonessential amino acids, 0.1 mM ß-mercaptoethanol, and 1% penicillin/streptomycin. After 7 days, we transferred the resultant embryoid bodies (EB) into six-well plates coated with Matrigel (8 EB per well) in differentiation medium that consisted of DMEM, 20% fetal bovine serum (FBS, Gibco, USA), 1% MEM non-essential amino acid solution, and 1% penicillin/streptomycin. The medium was changed every other day. 

### Real-time quantitative polymerase chain reaction

We performed real-time quantitative polymerase chain reaction (qRT-PCR) with a SYBR® Premix Ex TaqTM II Kit and Applied Biosystems 7500. Primers for *GAPDH, IL-1ß, IL-6, IL-8,* and *CCL2* were as previously reported (20). Relative transcription levels were determined using the 2^-ΔΔCT^ analysis method. 

## Results

### Isolation of peripheral blood mononuclear cells

**Fig.1:**
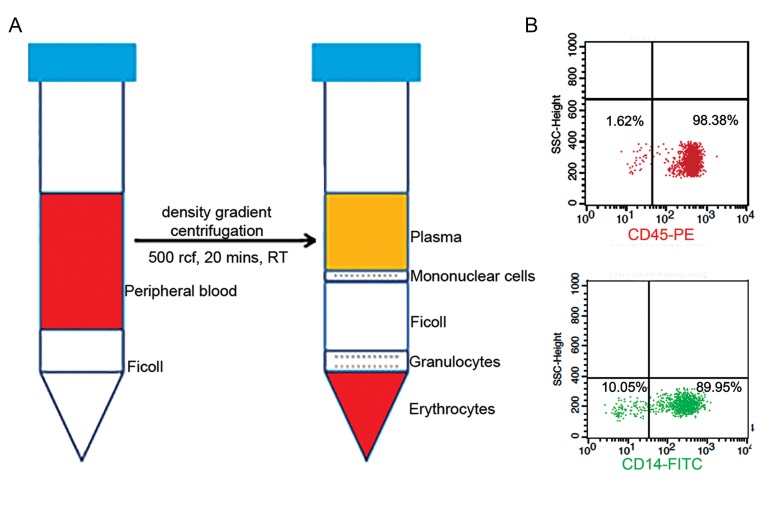
Experimental protocol for isolation of human peripheral blood mononuclear cells (PBMCs) from whole blood. **A.** Steps for isolation of human PBMCs from whole circulating blood by density gradient centrifugation and **B.** Flow cytometry analysis showed that the PBMCs significantly expressed the CD45^+^ or CD14^+^ genotypes.

PBMCs were purified from 7.5 mL of human whole circulating blood obtained from normal and SZ individuals by density centrifugation using a Ficoll gradient. This centrifugation separated the lymphocytes, monocytes, and plasma (Fig .1A). The PBMC layers were carefully transferred to a new tube and washed twice with 1X PBS. After centrifugation, the cells were resuspended in the appropriate volume of culture medium. Further, flow cytometry analysis showed that isolated PBMCs were mainly a subset of CD45^+^ or CD14^+^ monocyte cells (Fig .1B). The human PBMCs were then recovered with a final centrifugation and stored at -80oC. 

### Generation of induced pluripotent stem cells from peripheral blood mononuclear cells

**Fig.2:**
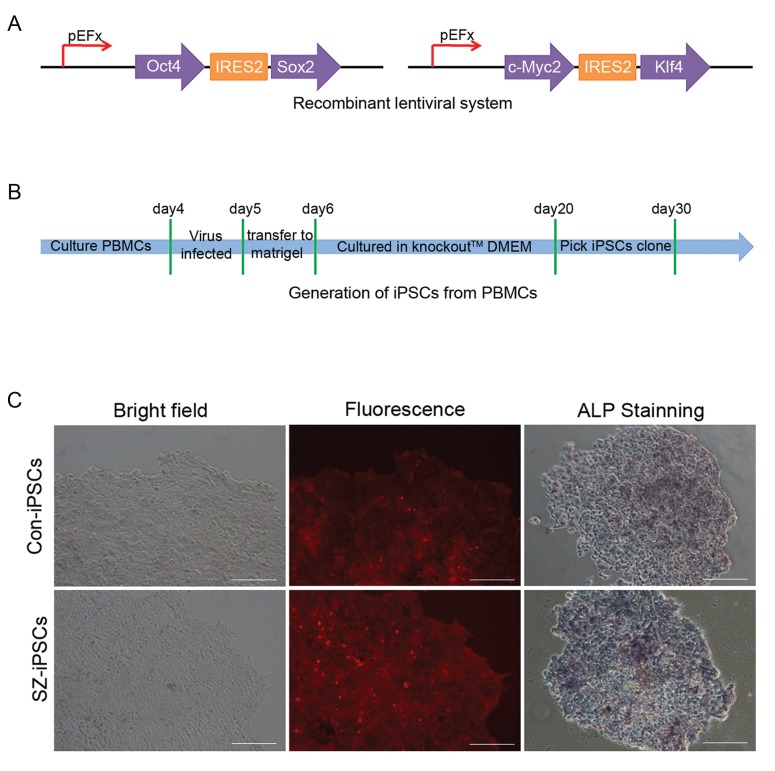
Generation of induced pluripotent stem cells (iPSCs) from peripheral blood mononuclear cells (PBMCs) using a recombinant lentivirus system. **A.** Recombinant lentivirus vectors that contained transcription factors Oct4, Sox2, Klf4, and c-Myc, **B.** Diagram of generation of iPSCs from PBMCs, and **C.** The morphology and alkaline phosphatase (ALP) staining of generated iPSCs from PBMCs in normal people and schizophrenia (SZ) individuals (scale bar: 50 μm).

Lentiviral vectors played a major role in the historical development of iPSC technology due to their ability to efficiently transduce murine cells for lasting expression of transgenes. The isolated PBMCs were transduced with a lentiviral plasmid that encoded OSKM factors (Fig .2A) and mCherry fluorescent protein at day 4. The OSKM factors in these PBMCs could be activated after they were infected by the virus. The cells were subsequently transferred to Matrigel and cultured with KnockoutTM DMEM medium. The iPSC-like colonies started to form 2 weeks later from the infected PBMCs that grew on the Matrigel (Fig .2B). We picked the colonies that displayed a typical morphology of iPSCs and mCherry protein expression from normal people and SZ patients (Fig .2C).

ALP enzymatic activity represents ESC pluripotency. We performed ALP staining and the results showed that the iPSC clones were ALP positive in both normal and SZ individuals (Fig .2C). These data showed that we could successfully establish iPSC clones with fewer transformed cells from the PBMCs in SZ patients.

### Characterization of induced pluripotent stem cells from peripheral blood mononuclear cells

**Fig.3:**
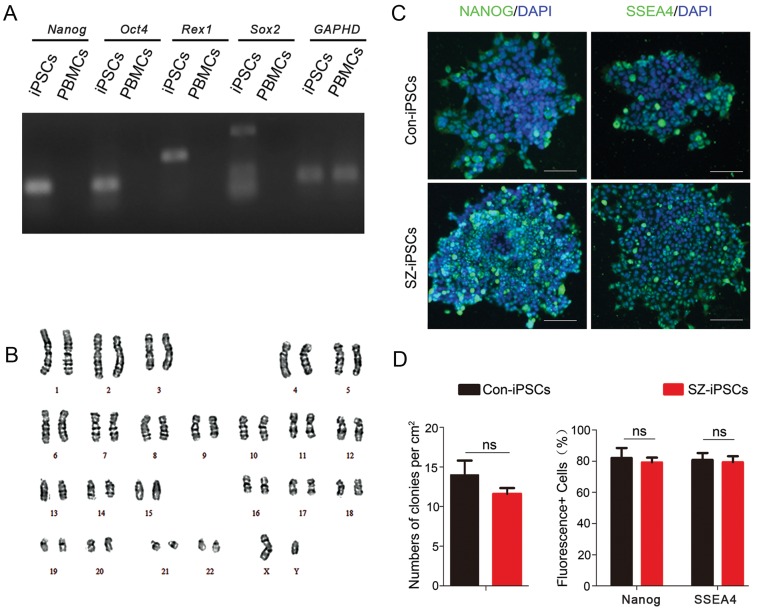
Characterization of induced pluripotent stem cells (iPSCs) from peripheral blood mononuclear cells (PBMCs). **A.** Reverse transcription polymerase chain reaction (RT-PCR) shows the different expressions of *Nanog, Oct4, Rex1,* and *Sox2* between iPSCs and PBMCs, **B.** Images for karyotyping of the schizophrenia (SZ)-iPSC line, **C.** Immunofluorescence staining of pluripotency markers Nanog and SSEA4 of expanded iPSCs fromPBMCs in normal individuals and SZ patients (scale bar: 50 µm), and D. Analysis of the numbers of iPSC clones per cm^2^ and fluorescent positive cellsby immunofluorescence staining. P values were determined by the student’s t test. ns; Not significant. Error bars indicate SEM.

We sought to assess whether the PBMCs from SZ patients could be reprogrammed into iPSCs. We selected the clones and re-plated them into six-well plates coated with Matrigel. We analyzed expressions of the pluripotency markers (*Nanog, Oct4, Rex1, Sox2*) in iPSCs and PBMCs by RT-PCR (Fig .3A). Karyotyping analysis showed that the iPSCs from SZ patients had normal karyotypes according to the standard G-banding technique (Fig .3B). We detected activity of pluripotency markers by immunostaining and observed relatively high expression levels of Nanog and SSEA4 in the generated iPSCs (Fig .3C). In addition, we analyzed the numbers of iPSC clones per cm^2^ and fluorescent cells in immunofluorescence staining (Fig .3D). There was no significant difference in iPSCs formed between normal people and SZ patients. These results indicated that the generated iPSCs from PBMCs displayed pluripotency as well as ESCs and other iPSCs.

**Fig.4:**
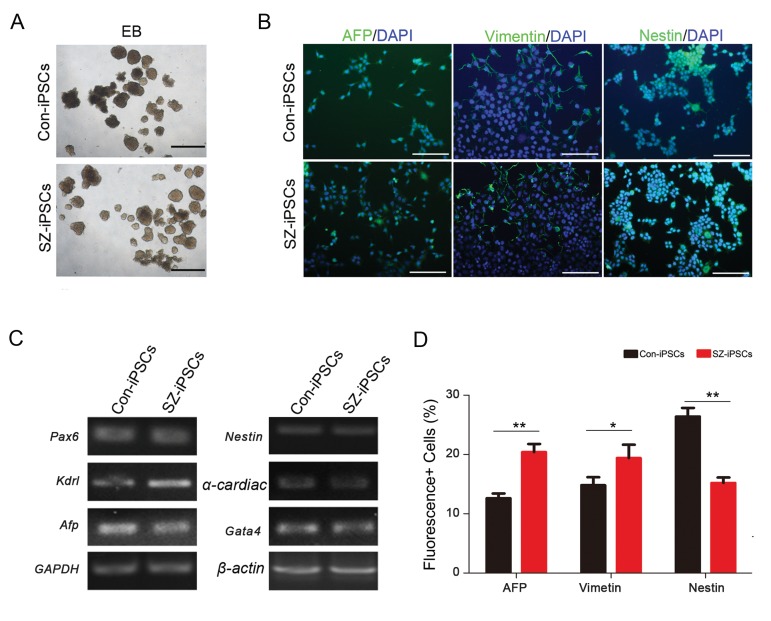
*In vitro* differentiation of schizophrenia induced pluripotent stem cells (SZ-iPSCs) from peripheral blood mononuclear cells (PBMCs). **A.** Embryoid body (EB) formation assay after 4 days of suspension culture, **B.** Reverse transcription polymerase chain reaction (RT-PCR) shows analogous expression levels of transcripts for the 3 germ-layers, **C.** Day 14 cultures contain cells immunoreactive for ectodermal (Nestin), mesodermal (Vimetin) and endodermal (AFP) germ layer markers (scale bar: 50 µm), and **D.** Analysis of fluorescent positive cells by immunofluorescence staining. P values are determined by the student’s t test. Error bars indicate SEM. *; P<0.05 and **; P<0.005.

To further determine the pluripotency of iPSCs, we cultured undifferentiated EBs (Fig .4A) with differential medium and then determined the differentiation potential of iPSCs by RT-PCR. The results showed both differentiated iPSCs from normal people (ConiPSCs) and SZ patients (SZ-iPSCs) expressed ectoderm (Pax6, Nestin), mesoderm (Kdrl, a-cardiac actin) and endoderm (Afp, Gata4) markers (Fig .4B). In addition, we performed *in vitro* differentiation assays and found that the iPSCs had the ability to differentiate into 3 germ layer-derived cell types. The assay used Nestin as a marker for ectoderm differentiation, Vimentin to mark mesoderm differentiation, and alpha fetoprotein (AFP) for endoderm differentiation (Fig .4C). However, a study of the fluorescent positive cells of Nestin, AFP, and Vimentin staining showed that the iPSCs had a weak capacity to differentiate into ectoderm and strong ability to differentiate into endoderm and mesoderm in SZ patients compared to normal people (Fig .4D). Taken together, the PMBCs of normal people and SZ patients had a similar ability to generate iPSCs. Nevertheless, it seemed that the iPSCs from SZ patients might have some defect for differentiating into ectoderm cells compared to those from normal people.

### Expression of inflammatory factors in induced pluripotent stem cells of normal individuals and schizophrenia patients 

**Fig.5:**
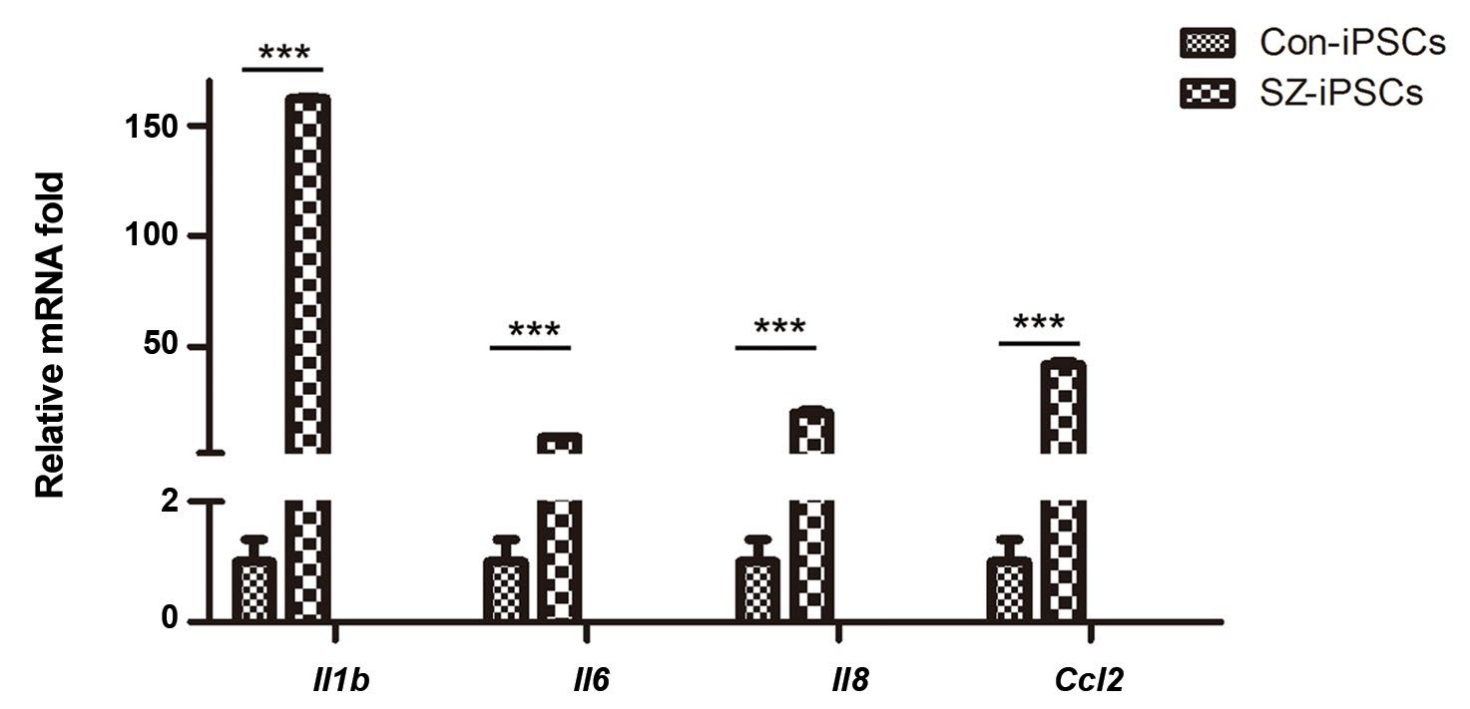
Expression of inflammatory factors between normal induced pluripotent stem cells (Con-iPSCs) and schizophrenia induced pluripotent stem cells (SZ-iPSCs). Real-time quantitative polymerase chain reaction (qRT-PCR) shows analogous expression levels of transcripts for IL-1b, IL-6, IL-8, and CCL2 between Con-iPSCs and SZ-iPSCs. P values are determined by the student’s t test. Error bars indicate SEM. ***; P<0.005.

There is ample evidence that inflammation/immune system can influence and shape the development of the CNS and behavior. The importance of this information as a factor in the etiology of SZ has been strongly emphasized by different laboratories that have investigated the effects of inflammation (21, 22). We detected the expression of inflammatory factors in iPSCs derived from normal and SZ patients when compared by qRT-PCR analysis. The results showed that the expression levels of *Il1b, Il6, Il8,* and *CCL2* in iPSCs from the SZ-iPSC group were much higher than the Con-iPSC group (Fig .5). This assay showed that the inflammation/immune system might play an important role in the etiology of SZ disorders. 

## Discussion

The appearance of human ESCs was believed to be beneficial in cell therapy and other medical applications (23). iPSCs, because of their similar characteristics to ESCs, became a novel alternative in stem cell biology. After the discovery of iPSCs, numerous scientists have established iPSCs from various somatic cell types, especially fibroblasts. Unlike fibroblasts, which are difficult to obtain, peripheral blood cells can be easily procured without the need for surgery (24). The biggest difference between fibroblasts and PBMCs is that the PBMCs first need to transform into an attached form during reprogramming. In our study, we have used Matrigel to help the floating cells attach and improve adherence and reprogramming. Then, the settled cells could expand into reprogrammed iPSCs. The pluripotency of the PBMC-derived iPSCs generated by our study could be used in therapeutic cell research and disease modeling.

SZ is a severe neurodevelopmental disorder that results from genetic and environmental factors. The pathogenesis of SZ is not fully understood, in part due to the limitations of current disease models and technology. In recent years, numerous researchers have focused on the establishment of an SZ disease model. The emergence of iPSC technology provides a new strategy to study SZ. Our study has demonstrated that PBMCs in SZ patients can be successfully reprogrammed into iPSCs without feeder layer cells, which are indispensable for reprogramming of fibroblasts. We can study the pathology and the mechanism of action of SZ using the SZ-iPSCs model *in vitro.*

Inflammatory factors play an important role ininfection and inflammation and are crucial mediators of the cross-talk between the brain and the immune system. SZ may be associated with an imbalance ininflammatory cytokines. Degradation products of inflammatory substances have been described in schizophrenic brain tissue (25). In terms of the cytokinepattern in SZ, interferon (IFN)-gamma, interleukin(IL)-2, soluble IL-2 receptors, IL-6, and IL-10 havebeen frequently observed in unmedicated SZ patients(26). However, expression of the inflammatory factorsof iPSCs from SZ patients remains unclear. Our findings from iPSC technology provide evidence of the establishment of an inflammatory syndrome in SZ.

## Conclusion

We successfully derived iPSCs from PBMCs of schizophrenic patients and showed that schizophrenic patients had a higher, stronger inflammatory response in iPSCs. Our results might lead to the development of develop new strategies for clinical diagnosis and treatment of SZ. 
